# Elucidating the pivotal molecular mechanisms, therapeutic and neuroprotective effects of lithium in traumatic brain injury

**DOI:** 10.1002/brb3.3595

**Published:** 2024-06-14

**Authors:** Seidu A. Richard

**Affiliations:** ^1^ Department of Medicine Princefield University Ho Ghana; ^2^ Institute of Neuroscience, Third Affiliated Hospital Zhengzhou University Zhengzhou China

**Keywords:** bipolar disorder, lithium, neuroinflammation, neuroprotection, neurotransmission, TBI

## Abstract

**Introduction:**

Traumatic brain injury (TBI) refers to damage to brain tissue by mechanical or blunt force via trauma. TBI is often associated with impaired cognitive abilities, like difficulties in memory, learning, attention, and other higher brain functions, that typically remain for years after the injury. Lithium is an elementary light metal that is only utilized in salt form due to its high intrinsic reactivity. This current review discusses the molecular mechanisms and therapeutic and neuroprotective effects of lithium in TBI.

**Method:**

The “Boolean logic” was used to search for articles on the subject matter in PubMed and PubMed Central, as well as Google Scholar.

**Results:**

Lithium's therapeutic action is extremely complex, involving multiple effects on gene secretion, neurotransmitter or receptor‐mediated signaling, signal transduction processes, circadian modulation, as well as ion transport. Lithium is able to normalize multiple short‐ as well as long‐term modifications in neuronal circuits that ultimately result in disparity in cortical excitation and inhibition activated by TBI. Also, lithium levels are more distinct in the hippocampus, thalamus, neo‐cortex, olfactory bulb, amygdala as well as the gray matter of the cerebellum following treatment of TBI.

**Conclusion:**

Lithium attenuates neuroinflammation and neuronal toxicity as well as protects the brain from edema, hippocampal neurodegeneration, loss of hemispheric tissues, and enhanced memory as well as spatial learning after TBI.

## Graphical abstract

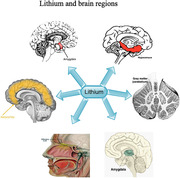

## 1 INTRODUCTION

Traumatic brain injury (TBI) refers to damage to brain tissue by mechanical or blunt force via trauma, and it is the prime cause of morbidity as well as mortality in young adults in developed as well as developing countries (Leeds et al., 2014; McGinn & Povlishock, 2016; Richard et al., 2017). TBI is often associated with compromised cognitive abilities, such as hitches in memory, learning, attention, as well as other higher brain function, that usually linger for years after the injury (Levin, 1998; Pierce et al., 1998). In terms of classification, TBI may be penetrating or non‐penetrating, diffuse or focal, vary in severity, location, as well as patient dynamics (Algattas & Huang, 2013).

TBI causes primary injuries such as mechanical damage to neurons, glia, and vascular structures, followed by secondary injuries such as excitotoxicity, oxidative stress, neuroinflammation, mitochondrial dysfunction, and axonal degeneration, and the latter is often associated with cognitive and behavioral dysfunction (Loane & Faden, 2010; F. Yu, Wang, et al., 2012). Also, multiple brain regions such as the cortex, hippocampus, and striatum show compromised neurotransmission that triggers alterations in cognitive function in experimental models of TBI and in the striatum of TBI patients (Donnemiller et al., 2000; Shin et al., 2011).

Lithium is an elementary light metal that is only used in salt form because of its high intrinsic reactivity (Haupt et al., 2021; F. Yu et al., 2013). Notably, for over 60 years now, it has been the mainstay of treatment for bipolar disorder (Haupt et al., 2021; F. Yu et al., 2013). It acts via multiple targets such as signaling proteins and organic cofactors as a free cation as well as anchoring to Na^+^ and Mg^2+^‐loaded nucleotides such as adenosine triphosphate (ATP) or guanosine triphosphate (GTP) (Dudev et al., 2019). Also, lithium attenuated several pathological processes such as apoptosis, oxidative stress, as well as mitochondrial and endoplasmic dysfunction, which are associated with the pathophysiology of TBI (Haupt et al., 2021).

Lithium is capable of ameliorating edema by restoring blood–brain barrier (BBB) distraction; averting inflammation via blockade of microglia activation as well as cyclooxygenase‐2 (COX‐2) stimulation; safeguarding neurons via inhibition of excess N‐methyl‐d‐aspartate (NMDA) receptor stimulation as well as calcium influx; stabilizing mitochondria through B‐cell lymphoma 2 (Bcl‐2)‐dependent mechanism; blocking mitochondrial release of cytochrome‐c (Cyto C) as well as apoptosis‐inducing factor; and blocking calpain as well as caspase‐3 stimulation (Bachmann et al., 2009; Q. Li et al., 2010).

Currently, although several basic and clinical research advances have been made in the use of lithium for the management of TBI, current reviews summarizing the molecular mechanisms induced by lithium following TBI are lacking. Thus, this review explores the pivotal molecular mechanisms and therapeutic and neuroprotective effects of lithium in TBI.

The “Boolean logic” was used to search for articles on the subject matter in PubMed and PubMed Central as well as Google Scholar with search terms like lithium and/or TBI, and brain regions, neurotransmission, neuroinflammation, signaling pathways, and neuroprotection were retrieved and discussed. Also, data on the pathophysiology of TBI were searched and discussed. Studies involving both humans and animals as well as both clinical research and basic research were critically reviewed. Articles that did not report or discuss interrelations between lithium and TBI mechanisms were excluded from this review.

## 2 LITHIUM ADMINISTRATION AND ADVERSE EFFECTS

Lithium is usually administered orally, either in the form of pills, capsules, or liquid, and typically requires about 1–3 weeks to manifest its effects, leading to symptom alleviation as well as remission (Oruch et al., 2014; Wen et al., 2019). However, lithium therapy is associated with adverse side effects that may manifest at any stage of the therapy (Ferensztajn‐Rochowiak & Rybakowski, 2023; Gitlin, 2016). Notably, only a few of these adverse effects have a substantial impact on the success of lithium therapy. Clinically, these side effects are categorized based on the organ or system, such as renal, neurological (cognitive), cardiologic, gastrointestinal, metabolic (thyroid), dermatological, and sexual (Ferensztajn‐Rochowiak & Rybakowski, 2023; Gitlin, 2016). The recognition and management of these adverse side effects often include watchful waiting in cases of tolerance and the use of antidotes for specific side effects. Also, modification of the drug's administration by lowering the dose leads to decrease in serum concentration. Furthermore, altering the time of administration by providing or switching to a different lithium formulation is often beneficial. Moreover, discontinuation or/and change to a different mood stabilizer is beneficial in rare cases (Ferensztajn‐Rochowiak & Rybakowski, 2023; Gitlin, 2016).

### 2.1 Brief pathophysiology of TBI

TBI is categorized into directly or indirectly based on mode of occurrence and penetrating or non‐penetrating after the traumatic process (Algattas & Huang, 2013; McGinn & Povlishock, 2016). Notably, primary TBI occurs as of the direct consequence of external mechanical forces such as acceleration and deceleration linear forces, rotational forces, forces triggered by blast winds related to blast injury, blunt impact, as well as penetration by projectile objects, leading to alteration of the brain tissue as well as disturbance of normal brain function (Algattas & Huang, 2013). Also, these forces directly injure the neurons, axons, dendrites, glia, as well as blood vessels in a focal, multifocal, or diffuse pattern and trigger a vigorous chain of multifaceted cellular, inflammatory, mitochondrial, neurochemical, as well as metabolic changes (Algattas & Huang, 2013; Loane & Faden, 2010; McKee & Daneshvar, 2015).

Distinctly, the classification of TBIs into focal, multifocal, or diffuse is often based on the pattern of lesion either localized or extend of spread (Algattas & Huang, 2013). Also, most TBIs are heterogeneous with both focal as well as diffuse components, although injuries are often considered primarily focal or diffuse (Dixon, 2017; Schmidt et al., 2004). Furthermore, focal injuries are associated with mass effect and include contusion, epidural hematoma, subdural hematoma, as well as intraparenchymal hemorrhage, while diffuse injuries are widely distributed in many anatomic regions and encompass axonal injury, hypoxic‐ischemic injury, as well as microvascular injury (Kabadi & Faden, 2014). Comparatively, the mortality rate for severe focal injuries is about 40%, while that for severe diffuse injuries is about 25% (Marshall et al., 1991).

Diffuse axonal injury (DAI), which triggers diffuse degeneration of cerebral white matter, diffuse vascular injury (DVI), and diffuse hypoxia/ischaemia are critical components of diffuse brain injury (Smith et al., 2003; Smith & Meaney, 2000). Also, the magnitude of axonal damage in DAI is very crucial in the outcome of TBI (McKee & Daneshvar, 2015). Furthermore, the primary swelling following TBI and further damage of injured axons often trigger alterations in ionic homeostasis. Moreover, osmotic swelling of axons facilitates Na^+^ influx, whereas upsurge in intracellular Ca^2+^ facilitates lethal induction of proteases resulting in further cytoskeletal damage as a result of these ionic changes (Blennow et al., 2012). Additionally, DVI is often associated with vascular congestion throughout the brain following the injury (Ekici et al., 2014).

**FIGURE 1:**
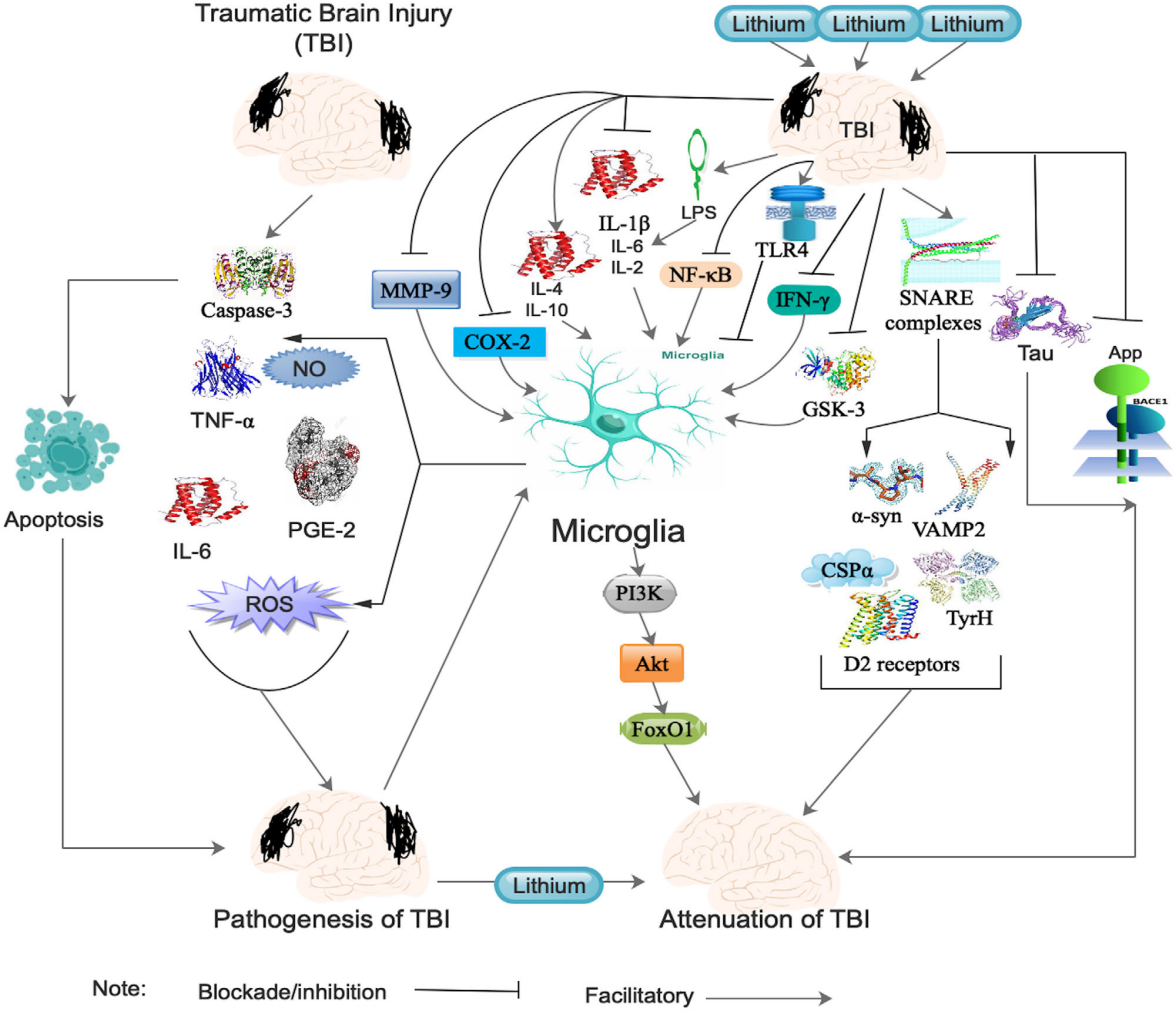
Shows the pathogenesis of TBI and the effect of lithium treatment on TBI‐associated neuroinflammation. Refer to the text for detailed explanations. APP, amyloid precursor protein; BACE1, β‐site APP‐cleaving enzyme 1; COX, cyclooxygenase; CSPα, cysteine string protein α; FoxO1, forkhead box protein O1; GSK‐3, glycogen synthase kinase 3; IFN‐γ, interferon gamma; IL, interleukin; LPS, lipopolysaccharide; MMP‐9, matrix metallopeptidase‐9; NF‐κB, nuclear factor‐κB; NO, nitric oxide; PGE‐2, prostaglandin E2; PI3K, phosphoinositide 3‐kinase; ROS, reactive oxygen species; SNARE, soluble N‐ethylmaleimide‐sensitive factor activating protein receptor; TLR4, toll‐loke receptor 4; TNF‐α, tumor necrosis factor‐alpha; TyrH, tyrosine hydroxylase; VAMP2, vesicle‐associated membrane protein 2; α‐syn, α‐synuclein.

DVI triggers a breakdown of the BBB accompanied by an inflammatory response characterized by infiltration of neutrophils as well as macrophages, activation of glial cells, and upregulation of the secretion of pro‐inflammatory cytokines (Figure 1) (Dixon, 2017; Schmidt et al., 2004). Also, astrocytes, which are the most copious nonneuronal cell type in the brain, are sources of pro‐inflammatory cytokines, which progresses from glial scar inhibition to neural regeneration in the advanced stages of injury (Ekici et al., 2014). Moreover, the conversion of glial cells to their “reactive” state and the concomitant upsurge in secretion of cytokines as well as chemokines trigger neurodegeneration following the injury (G. Chen et al., 2008).

Predominantly, surviving astrocytes in the injured region begin to exhibit hypertrophy as well as proliferation, which is referred to as “reactive astrogliosis” within a few hours of practically any type of brain injury (Ekici et al., 2014). Also, this response stimulated the migration of microglia as well as macrophages to the injured area (Williams et al., 2007). Also, reactive astrocytes augment the secretion of glial fibrillary acidic protein (GFAP), a structural protein that is usually utilized as astrocyte markers (Myer et al., 2006). Moreover, hypertrophic astrocytes with conspicuous immunoreactive processes are often diffusely disseminated throughout the region of injury and around the cell body (Fix et al., 1996). Furthermore, reactive astrocytes interlink their processes to form a barrier called “anisomorphic gliosis” at the immediate location of injury, and this glial scar often impairs the regeneration of axons (Das et al., 2012; C.‐H. Yu et al., 2011).

Secondary brain injury arises as a hindrance of the primary brain injury and cascade of events includes ischemic as well as hypoxic damage, cerebral edema, raised intracranial pressure, hydrocephalus, and infection (Algattas & Huang, 2013; Loane & Faden, 2010; McKee & Daneshvar, 2015). Specifically, cellular as well as vasogenic fluid buildup in the brain resulting in cerebral edema, raised intracranial pressure, as well as cerebral ischemia within hours of the trauma (McKee & Daneshvar, 2015). Also, brain dysfunction as well as morbidity are further augmented by a decrease in cerebral blood flow or oxygen concentration below a threshold level or via cerebral herniation (McKee & Daneshvar, 2015). Moreover, at the molecular level, secondary brain injuries trigger oxidative stress via free radical formation as well as lipid peroxidation, excitotoxicity via excess glutamate release, and augmented NMDA receptor stimulation that may result in increased calcium ion influx (Algattas & Huang, 2013; G. Chen et al., 2008; Das et al., 2012; Leeds et al., 2014).

Additionally, secondary brain injuries trigger neuroinflammation via proinflammatory cytokines, nitric oxide, or prostaglandins; mitochondrial distraction associated with augmented poly (ADP‐ribose) polymerase 1 (PARP‐1) stimulation, reduced NAD^+^/ATP levels, augmented calpain stimulation, as well as permeabilization of mitochondrial permeability transition pore (mPTP); failure of the BBB associated with cerebral edema, hypoxia, as well as ischemia; and cellular death via necrosis, caspase‐dependent like caspase‐3 apoptosis, and caspase‐independent or apoptosis inducing factor (Figure 1) (Algattas & Huang, 2013; G. Chen et al., 2008; Das et al., 2012; Leeds et al., 2014). It is worth noting that these secondary events are often reversible in mild cases but severe cases may lead to neurological sequelae like neuropsychiatric disturbances such as depression, anxiety, bipolar disorders, and other posttraumatic stress disorders as well as behavioral and cognitive deficits (Leeds et al., 2014).

### 2.2 Lithium and brain regions

**FIGURE 2:**
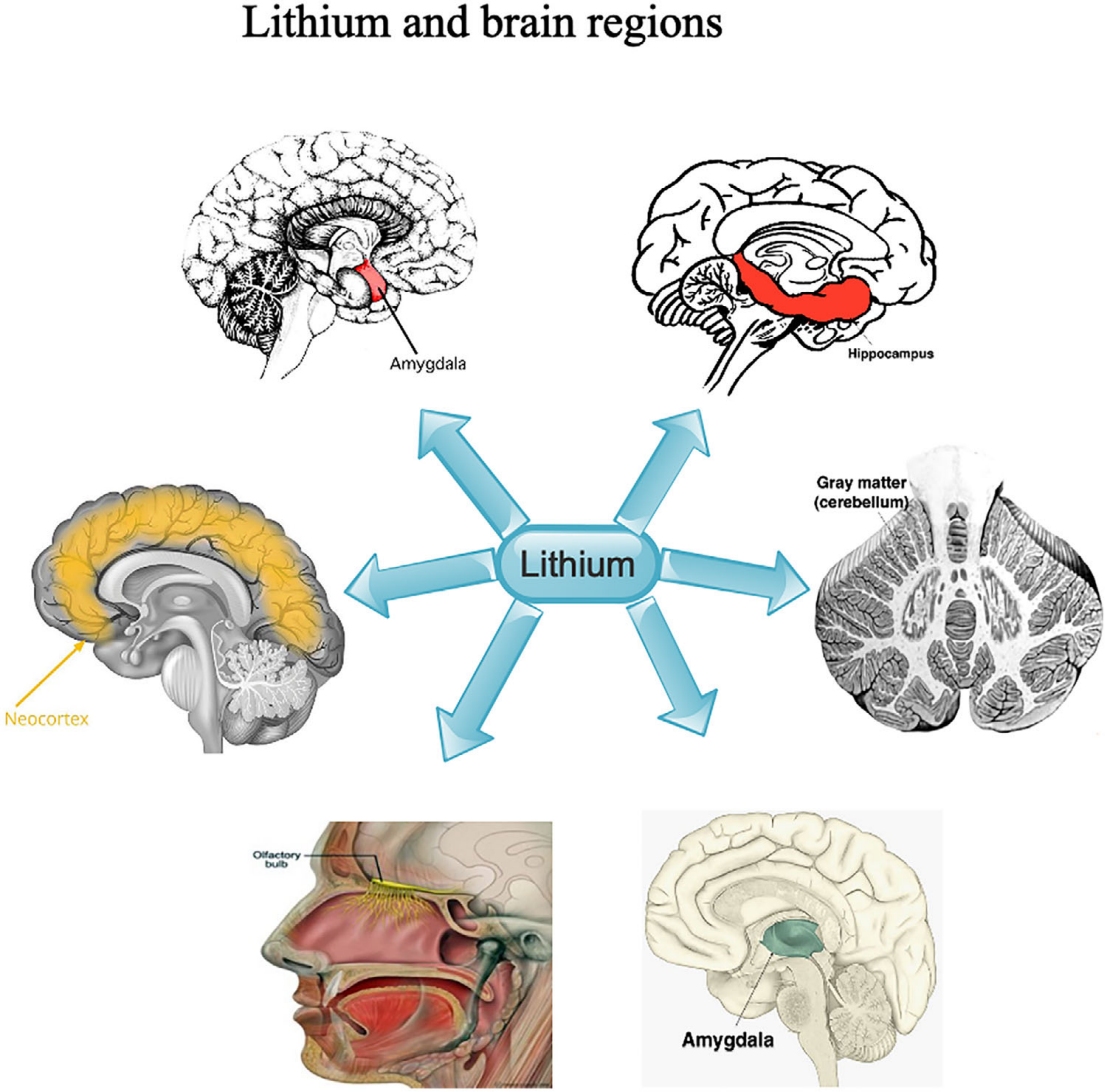
Show the key brain regions lithium influences. Note: Colors are used to show the regions in the diagram.

Lithium has been detected in almost all brain regions, mainly in neurogenic brain regions, but the highest lithium concentrations were detected in the hippocampus, thalamus, neo‐cortex, olfactory bulb, amygdala, and gray matter of the cerebellum (Figure 2) (Thellier et al., 1980; Zanni et al., 2017). Also, the hippocampus is the brain area that controls learning, memory, cognition, as well as mood, and smaller hippocampal volumes have been implicated in psychiatric disorders (Leuner & Gould, 2010). Furthermore, the mammalian hippocampus is proficient in neurogenesis even in adulthood, with about 700 new neurons being generated each day in the human dentate gyrus (DG), unlike most parts of the brain (Spalding et al., 2013). Moreover, augmentation in density of neurons, glia, as well as hippocampal volume, which is correlated with enhanced mood as well as cognition, was observed following lithium treatment (Chiu et al., 2013).

Notably, augmented hippocampal atrophies were detected in both hemispheres mainly after treatment with low‐dose lithium, which correlated with decreased cell proliferation as well as neurogenesis in the hippocampus (Palmos et al., 2021). Also, it was further observed that chronic lithium treatment augmented substance P's concentration of the dopamine‐associated brain areas like the substantia nigra, the frontal cortex, as well as the nucleus accumbens but not the hypothalamus, hippocampus, or brain stem (Le Douarin et al., 1983). Furthermore, high‐dose lithium augmented the size of the ipsilateral hemisphere of the striatum as well as the corpus callosum areas but not the hippocampus (Ciftci et al., 2020). Moreover, high‐dose lithium expressively decreased infarct volume and improved atrophies in the striatum as well as corpus callosum, although low‐dose lithium had no significant protective effect following brain injury (Ciftci et al., 2020).

In TBI, lithium attenuated neuronal degeneration in the hippocampal DG, and the apparent anxiolytic as well as antidepressant‐like effects of lithium stem from its capacity to attenuate neurodegeneration in this brain region (Ciftci et al., 2020). Also, augmented Aβ load was associated with massive hippocampal neuronal death as well as cognitive impairment in a TBI model (Smith et al., 1998). Furthermore, inhibition of Aβ levels had beneficial effects against TBI because blockade of β‐ or c‐secretase enzymes, which stimulate the production of Aβ from amyloid precursor protein (APP) augmented hippocampal tissue conservation as well as enriched functional outcome (Loane et al., 2009).

Post‐injury treatment with lithium attenuated TBI‐induced Aβ load upsurge, APP buildup in axonal bulbs, as well as β‐site APP‐cleaving enzyme 1 (BACE1) oversecretion in the hippocampus and corpus callosum areas enriched in APP as well as Aβ (Figure 1) (F. Yu, Zhang, et al., 2012). Thus, lithium attenuated APP accumulation in axonal bulbs after TBI as well as reduced the availability of substrate necessary to produce Aβ (F. Yu, Zhang, et al., 2012). Also, the thalamus is associated with memory and patients with thalamic injuries exhibit high memory impairments as well as spatiotemporal relations (Weiler et al., 2011). Furthermore, augmented thalamic inflammation is correlated with severity of cognitive function in TBI patients (Ramlackhansingh et al., 2011). Moreover, TBI‐induced augmentation of Tau phosphorylation in the thalamus was also attenuated by lithium 3 days after the injury (Figure 1) (F. Yu, Zhang, et al., 2012). Additionally, lithium‐attenuated TBI‐induced Tau phosphorylation was detected at Thr205 in the thalamus. Furthermore, lithium augmented hippocampal tissue conservation in TBI model mice (F. Yu, Zhang, et al., 2012).

TBI expressively attenuated the abundance of multiple monomer‐soluble N‐ethylmaleimide‐sensitive factor activating protein receptor (SNARE) proteins as well as SNARE complexes in the hippocampus, and these modifications were associated with changes in synaptic vesicle density as well as distribution 1 week after controlled cortical impact (CCI) injury (Carlson et al., 2017, 2016). Thus, SNARE proteins as well as SNARE complex formation were attenuated in striatal synapses after TBI. Also, following TBI, lithium treatment augmented the abundance of several SNARE proteins, such as α‐synuclein (α‐syn) and vesicle‐associated membrane protein 2 (VAMP2), as well as induced SNARE complex formation in the hippocampus at multiple time‐points post‐injury (Figure 1) (Carlson et al., 2017). Additionally, lithium treatment augmented the copiousness of α‐syn, cysteine string protein α (CSPα), phosphorylated tyrosine hydroxylase (TyrH), as well as D2 receptors in striatal synaptosomes following TBI (Figure 1) (Carlson & Dixon, 2018). Moreover, CSPα was decreased within 1 day after TBI and preceded decreases in SNARE complex formation following lithium treatment (Carlson et al., 2016).

### 2.3 Lithium and neurotransmission

Neurotransmission is a process by which neurons pass information to each other, and these signals are passed from one neuron to the next at synapses. Neurotransmitters drift across the synaptic cleft until they reach the outer surface of the dendrite, the postsynaptic density, after being released from an axon terminal. Many substances, such as amino acids, gases, small organic chemicals, as well as short peptides, act as neurotransmitters (Guerriero et al., 2015). Also, neurotransmission impairments are associated with modifications in intrasynaptic vesicular protein mechanism essential for translocation as well as docking of neurotransmitter‐containing vesicles at the active zone of the synapse (Söllner et al., 1993). Furthermore, alterations in neurotransmitter concentrations, receptor populations, as well as specific cell survival are important contributing factors (Carlson & Dixon, 2018).

Glutamate is the primary excitatory neurotransmitter in the brain, whereas *γ*‐aminobutyric acid (GABA) is the primary inhibitory neurotransmitter (Guerriero et al., 2015). Thus, the balance of glutamatergic and GABAergic tone is fundamental for normal neurologic function. It is worth noting that acute posttraumatic glutamate secretion is associated with excitotoxicity following TBI, which triggers neuronal injury, cell death, as well as dysfunction of surviving neurons, while delayed disruption of excitatory glutamate circuits triggers deficits in cognitive as well as motor function in experience‐dependent plasticity (Guerriero et al., 2015). Also, pyramidal neurons, situated in the cortex as well as the hippocampus of mammals, and neurons of the midbrain, hypothalamus, as well as cerebellum produce glutamate that is essential for excitatory signaling pathways (Spruston, 2008).

Notably, an upsurge in extracellular glutamate was detected 24 h after TBI, which persisted for as long as 4 days and was directly correlated to posttraumatic mortality (Chamoun et al., 2010). Additionally, an upsurge in extracellular glutamate was observed 1 h after TBI, with a much more acute fluid percussion injury (FPI) after a CCI in a rodent model during microdialysis analysis (Folkersma et al., 2011; Katayama et al., 1990). Also, in humans, a reduction in glutamate levels was observed for 1–6 days in the motor cortex but not in the dorsal lateral prefrontal cortex or hippocampi and returned to baseline in the chronic phase of injury at 6 months (Henry et al., 2010). Furthermore, pretreatment with lithium protected cultured neurons from glutamate‐induced cell death as well as decreased the oxidative stress associated with neuropeptide S (Table 1) (Castro et al., 2009; Hashimoto et al., 2002).

**TABLE 1:** Show the most commonly neurotransmitters and the mechanisms via which lithium influence these neurotransmitters to achieve neurotransmission in traumatic brain injury (TBI). Studies on effects of lithium and neurotransmitter not mentioned here on TBI are warranted.

Neurotransmitter	Type	Mechanisms via which lithium influence neurotransmission in TBI	Reference
Glutamate	Excitatory	Pretreatment with lithium protected cultured neurons from glutamate‐induced cell death as well as decreased the oxidative stress associated with neuropeptide S.	(Castro et al., 2009; Hashimoto et al., 2002)
		Lithium was capable of attenuating glutamate excitotoxicity, oxidative stress, NMDA receptor activation as well as Ca^2+^ influx.	(Basselin et al., 2006; Nonaka et al., 1998)
		Glutamate transmission was restored via direct downregulation of NMDA receptor as well as augmentation of glutamate reuptake following chronic lithium treatment.	(Malhi et al., 2017)
		Lithium is capable of ameliorating injured excitotoxic effects of Ca^2+^ influx via NO‐nitrosative pathway since it capable of downregulating glutamate neurotransmission at the NMDA receptor.	(Munteanu et al., 2022; Nonaka et al., 1998)
γ‐Aminobutyric acid (GABA)	Inhibitory	Lithium augmented the concentration of GABA in the plasma as well as CSF following brain injury.	(Vargas et al., 1998)
		Lithium's effects on GABA facilitated the secretion of neuroprotective proteins, and an upsurge in GABA, in response to lithium, decreased the concentration of glutamate, which further downregulates NMDA receptor activity.	(Ghasemi & Dehpour, 2011)
Dopamine	Excitatory and inhibitory	Lithium treatment expressively augmented P‐Ser40 TH as well as the levels of D2 receptor, signifying that lithium stimulated dopamine synthesis as well as neurotransmission.	(Carlson & Dixon, 2018)
		Lithium‐induced augmentation of *α*‐syn after TBI triggered differential effects on the dopaminergic system.	(Carlson & Dixon, 2018)
		Lithium enhanced K^+^ evoked dopamine neurotransmission in the striatum at 1 week postinjury.	(Ferrie et al., 2005)
		Lithium is capable of influencing the dopaminergic pathways by normalizing presynaptic neurotransmission as well as postsynaptic activities.	(Ichikawa et al., 2005)
		Chronic lithium treatment modified the function of G‐protein active as well as inactive its subunits resulting in the modulation the transduction mechanisms.	(Manji & Lenox, 2000)

Abbreviations: CSF, cerebrospinal fluid; NMDA, N‐methyl‐D‐aspartate; NO, nitric oxide; α‐syn, α‐synuclein.

Lithium was capable of attenuating glutamate excitotoxicity, oxidative stress, NMDA receptor activation, as well as Ca^2+^ influx (Table 1) (Basselin et al., 2006; Nonaka et al., 1998). Also, lithium selectively competes with Mg^2+^ at binding sites on NMDA glutamate receptors, leading to acute stimulation, which in turn augments the availability of glutamate in the postsynaptic neuron (Hokin et al., 1996; C.‐T. Li et al., 2019). Moreover, glutamate transmission was restored via direct downregulation of the NMDA receptor as well as augmentation of glutamate reuptake following chronic lithium treatment (Table 1) (Malhi et al., 2017). Thus, lithium is capable of ameliorating the injured excitotoxic effects of Ca^2+^ influx via nitric oxide (NO)‐nitrosative pathway since it is capable of downregulating glutamate neurotransmission at the NMDA receptor (Table 1) (Munteanu et al., 2022; Nonaka et al., 1998).

Alternatively, GABA is generated by interneurons that regulate cortical as well as thalamocortical circuits that transmit sensory information as well as coordinate motor functions, attention, and memory (Castro‐Alamancos & Connors, 1997). Also, GABA controls excitatory pathways in the brain. However, following injury, loss of GABA‐generating cells disrupts the balance of excitation as well as inhibition resulting in further cell injury as well as apoptosis (Guerriero et al., 2015). Furthermore, inhibiting GABA‐A receptors acutely triggered seizures in rats following lateral FPI as well as a more obvious structural damage, signifying the key role of GABA signaling in neuronal health following acute brain injury (Bao et al., 2011). Moreover, acute difference in the expression of GABA‐B subunit was observed in TBI models. Additionally, a subunit like α1/γ2, which is responsible for phasic inhibition, was downregulated following TBI, whereas α4/δ1, which is responsible for tonic inhibition, was upregulated (De Beaumont et al., 2012).

It is worth noting that changes in GABA‐A subunit expression were associated with glutamate‐induced excitatory signal, and GABA α1/γ2 subunit expression was augmented hours after diffuse FPI in rats but reduced by 24 h (Guerriero et al., 2015). Also, accumulation of cellular damages and compensatory alterations triggers imbalance of excitation as well as inhibition, resulting in post‐traumatic seizures or neurocognitive as well as behavioral alterations in the chronic stages of TBI (Guerriero et al., 2015). It was observed that the early phases of this imbalance in glutamate as well as GABA triggers mechanisms resulted in post‐traumatic epilepsy (Malhi et al., 2017). Furthermore, lithium augmented the concentration of GABA in the plasma as well as cerebrospinal fluid (CSF) following brain injury (Table 1) (Vargas et al., 1998). Moreover, lithium's effects on GABA facilitated the secretion of neuroprotective proteins, and an upsurge in GABA, in response to lithium, decreased the concentration of glutamate, which further downregulates NMDA receptor activity (Table 1) (Ghasemi & Dehpour, 2011).

Notably, the highly preserved SNARE complex and associated proteins such as CSPα and α‐syn are essential for the regulation of fusion of synaptic vesicles with the plasma membrane and the consequent release of neurotransmitters into the synaptic cleft (Carlson et al., 2017; Söllner et al., 1993). Also, CSPα and α‐syn are critical for the formation of the SNARE complex (Sharma et al., 2011). Mechanistically, lithium augmented the release of the SNARE complex and chaperone CSPα and α‐syn in in vitro and in vivo models of TBI (Cordeiro et al., 2003, 2000).

Additionally, an acute upsurge in dopamine concentration was detected within hours of TBI, followed by decreased dopamine neurotransmission weeks after injury in experimental TBI models (Massucci et al., 2004; Wagner et al., 2009). Furthermore, changes in dopamine signaling, axonal injury, impaired mitochondrial function, as well as neuronal loss in the striatum or substantia nigra were observed a few days to weeks post‐injury in experimental TBI models (Hutson et al., 2011). Moreover, TBI‐induced alterations in TyrH activity, dopamine transporter secretion, as well as dopamine receptor were detected in the striatum weeks after injury, which was associated with the impairment of neurotransmission (Massucci et al., 2004; Wagner et al., 2009). Also, lithium treatment expressively augmented P‐Ser40 TyrH as well as the levels of D2 receptor, signifying that lithium stimulated dopamine synthesis as well as neurotransmission (Table 1) (Carlson & Dixon, 2018).

Furthermore, lithium augmented the levels of α‐syn as well as enhanced the phosphorylation of serine 40 of TyrH (Carlson & Dixon, 2018). Thus, lithium‐induced augmentation of α‐syn after TBI triggered differential effects on the dopaminergic system (Table 1). It is worth noting that CCI triggered impairment of evoked striatal dopamine neurotransmission weeks after TBI (Shin et al., 2011; Wagner et al., 2009). Additionally, CCI attenuated medial forebrain‐induced release as well as clearance of dopamine using fast scan cyclic voltammetry at 2 weeks post‐injury (Wagner et al., 2009). Besides, TBI generated a substantial decrease in dopamine release over a 40‐min period of high K^+^ CSF infusion using a similar microdialysis infusion strategy (Shim et al., 2012; Shin & Dixon, 2011). Notably, lithium enhanced K^+^‐evoked dopamine neurotransmission in the striatum at 1 week postinjury (Table 1) (Ferrie et al., 2005). Moreover, 25 days of lithium therapy triggered K^+^‐evoked dopamine secretion in the nucleus accumbens and persists for days after cessation of lithium (Ferrie et al., 2005).

Lithium attenuated extracellular dopamine resulting in a reduced reactivity to harmful stimuli in animal models (Ichikawa et al., 2005). Also, lithium is capable of influencing the dopaminergic pathways by normalizing presynaptic neurotransmission as well as postsynaptic activities (Table 1) (Ichikawa et al., 2005). Mechanistically, G‐protein‐coupled dopamine receptors triggered cellular signal transduction mechanisms resulting in the stimulation of a cascade of activities that regulate dopamine neurotransmission (Malhi et al., 2017). Furthermore, chronic lithium treatment modified the function of G‐protein active as well as inactive subunits, resulting in the modulation of transduction mechanisms (Table 1) (Manji & Lenox, 2000). Studies on the effects of lithium on other neurotransmitters not captured in this review in TBI are needed.

### 2.4 Lithium and inflammation

Neuroinflammation refers to inflammatory response within the brain or spinal cord, which is mediated by the generation of cytokines, chemokines, reactive oxygen species (ROS), as well as secondary messengers (DiSabato et al., 2016). These intermediaries are generated by resident central nervous system (CNS) glia such as microglia and astrocytes, endothelial cells, as well as peripherally derived immune cells, which triggers immune, physiological, biochemical, as well as psychological consequences as a result of these neuroinflammatory responses (DiSabato et al., 2016). It is well established that neuroinflammation is key to the pathogenesis of TBI (Loane & Faden, 2010). Also, neutrophils, macrophages, as well as stimulated microglia act as scavenger cells to eradicate cellular debris as well as release cytotoxic or neurotrophic molecules into the injured tissue (Schmidt et al., 2004; Williams et al., 2007; C.‐H. Yu et al., 2011). Furthermore, lithium also suppresses neuroinflammation as well as decrease neuronal toxicity resulting in neuronal survival (F. Yu, Wang, et al., 2012).

Microglia are very critical in triggering inflammatory response to injury or infection in the brain and stimulated microglia trigger several immune effector functions normally associated with macrophages (Yuskaitis & Jope, 2009). Also, when microglia are activated via anomalous stimulations, like neurotoxins, neuronal debris, as well as injury, they trigger numerous inflammatory mediators such as tumor necrosis factor‐alpha (TNF‐α), prostaglandin (PG)‐E2, interleukin (IL)−6, NO, and ROS (Figure 1) (Dong et al., 2014). Moreover, build up of these pro‐inflammatory as well as cytotoxic intermediaries is detrimental to the neurons and consequently triggers more activation of microglia in a vicious cycle (Figure 1) (Herrera et al., 2005). Furthermore, microglia have been isolated in areas surrounding injury sites from 3 days to 8 weeks after TBI (I. Yu et al., 2010).

Additionally, lithium regulated inflammation as well as attenuated inflammation‐induced neurotoxicity via the attenuation of microglial migration (Beurel et al., 2010; Yuskaitis & Jope, 2009). Also, lithium blocked the secretion of toll‐loke receptor 4 (TLR4), which was associated with the repressive effect of lithium on microglial activation. Furthermore, lithium blocked lipopolysaccharide (LPS) stimulated upregulation of TLR4 in microglia via the stimulation the phosphoinositide 3‐kinase (PI3K)/protein kinase B (PKB, or Akt)/forkhead box protein O1(FoxO1) (PI3K/Akt/FoxO1) signaling pathway (Figure 1) (Dong et al., 2014). Moreover, attenuation of microglia activation via blockade of IL‐1β as well as histone deacetylase facilitated lithium neuroprotective effect against TBI (Figure 1) (Clausen et al., 2009). Furthermore, lithium blocked LPS‐stimulated activation of primary microglia as well as attenuated IL‐6 production from activated microglia (Figure 1) (Dong et al., 2014). Nevertheless, lithium triggered an upsurge in concentrations of IL‐4 and IL‐10, and a decline in concentrations of IL‐2 and interferon gamma (IFN‐γ) in healthy volunteers (Figure 1) (Guloksuz et al., 2012).

Notably, IL‐1β levels correlated with intracranial pressure following TBI (Hayakata et al., 2004). Also, IL‐1β neutralization triggered a reduced hemispheric tissue loss as well as mitigated cognitive deficits following TBI in mice, signifying that IL‐1β is crucial factor in post‐injury inflammatory response (Figure 1) (Clausen et al., 2009). Furthermore, chronic pre‐ and post‐injury lithium treatment attenuate concentrations of IL‐1β, as well as improved Morri's water maze performance of mouse TBI models (Zhu et al., 2010). It is worth noting that TNF‐α has been implicated in the generation of cerebral edema as well as secondary neuronal loss after TBI. Moreover, TNF‐α induced ischemic brain damage via modifications of BBB permeability, stimulation of microglia as well as astrocytes, and stimulation of cellular adhesion molecule secretion as well as recruitment of neutrophils (Gong et al., 1998).

Also, elevated concentration of TNF‐α was discovered in CSF as well as serum from patients with TBI (S.‐F. Chen et al., 2007). Similarly, cortical concentration of TNF‐α was expressively stimulated 24 h after TBI (Ekici et al., 2014). Also, lithium was able to attenuate TNF‐𝛼 concentrations under multiple experimental conditions (Nassar & Azab, 2014). Specifically, lithium blocked LPS‐stimulated activation of TNF‐α production from activated microglia following brain injury. It was further observed that an inverse correlation between lithium response and TNF‐𝛼 concentration to induce long‐term mood stabilizing (Guloksuz et al., 2012). Further studies involving the effect lithium has on TNF‐𝛼 in TBI models are needed. Moreover, COX, which is limiting enzyme responsible for the generation of PGs, has been implicated in neuroinflammation (Andreasson, 2010). Also, COX‐2, which is an inducible form of COX, is secreted by glutamatergic neurons in normal conditions and it was obviously upregulated in animal models of TBI (Ahmad et al., 2008). Additionally, lithium inhibited COX‐2 secretion in the cortex of TBI mice signifies its capacity of inhibiting neuroinflammation after TBI (Figure 1) (F. Yu, Wang, et al., 2012).

Matrix metallopeptidase‐9 (MMP‐9), a fundamental form of gelatinase, is able to degrade extracellular matrix, tight‐junction proteins, as well as augmentation of BBB permeability (Rosell et al., 2008). MMP‐9 gene promoter contains putative nuclear factor‐κB (NF‐κB) p65 binding sites and blockade of NF‐κB following ischemic injury inhibited MMP‐9 gene secretion (Van den Steen et al., 2002). It is worth noting that lithium was also capable of maintaining BBB integrity via the blockade of MMP‐9 expression (Figure 1) (Leeds et al., 2014; Van den Steen et al., 2002). Also, lithium triggered suppression of neuroinflammation by blocking stimulation of NF‐κB, which in turn decreased the upregulation of MMP‐9 as well as distraction of the BBB following TBI (Figure 1) (Van den Steen et al., 2002).

Glycogen synthase kinase 3 (GSK‐3), a regulator of glycogen metabolism, has been implicated in the immune regulation responses in the CNS (Beurel et al., 2010; King et al., 2014). GSK‐3 exists in two structurally analogous isoforms such as α and β (Woodgett, 1990). Also, GSK‐3β as well as Akt phosphorylation levels were augmented after TBI (Shapira et al., 2007). It is worth noting that lithium improved functional outcomes on multiple behavioral tests via blockade of GSK‐3β activity following TBI. Furthermore, lithium blockade effect on GSK‐3 resulted in decreased microglial migration, cytokine release, as well as neurotoxicity (Figure 1) (Yuskaitis & Jope, 2009).

### 2.5 Signaling mechanisms

**FIGURE 3:**
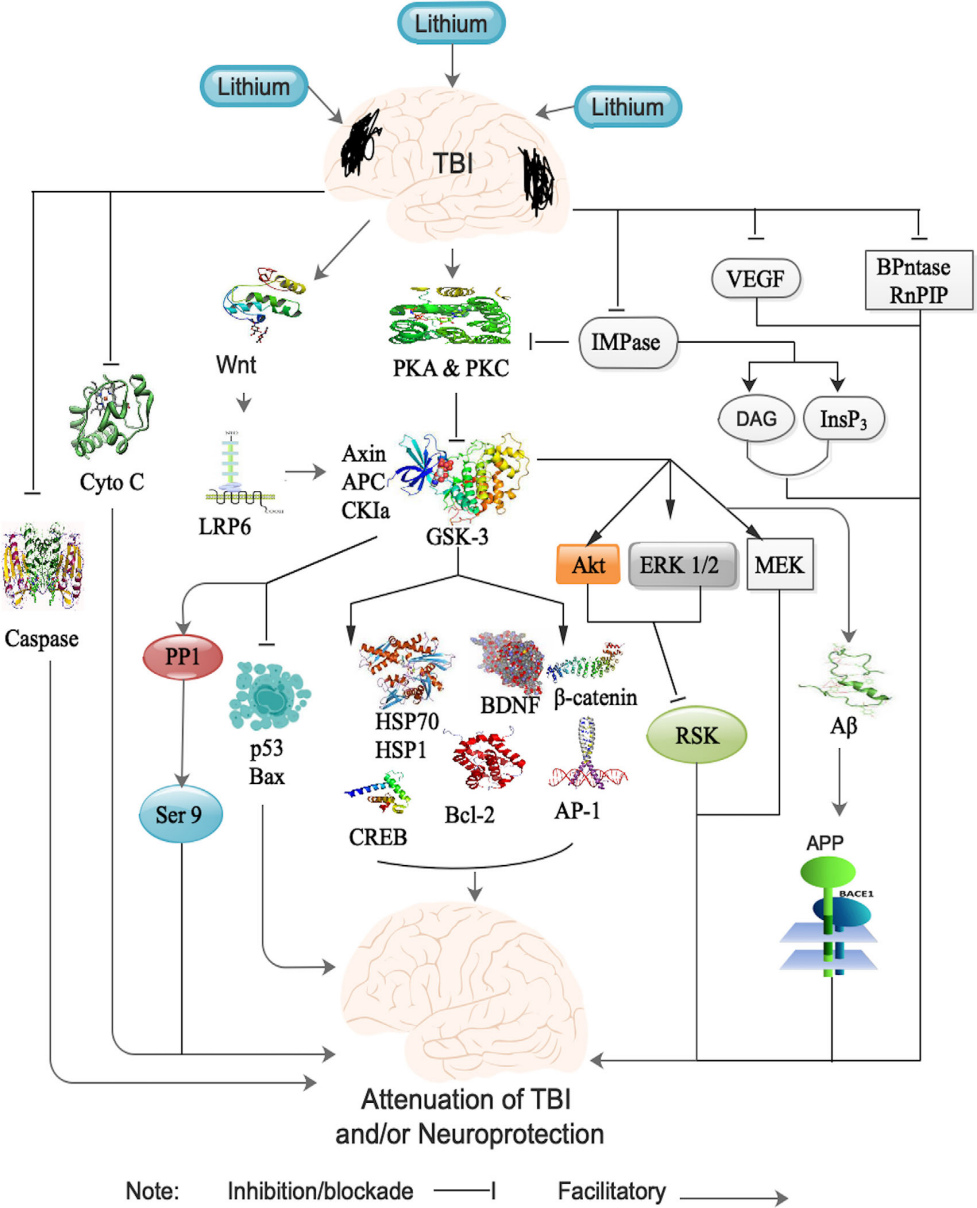
Show the key signaling pathways via which lithium triggers attenuation of TBI and/or neuroprotection. Refer to the text for detailed explanations. AP‐1, activator protein 1; APC, adenomatous polyposis coli; APP, amyloid precursor protein; Bcl‐2, B‐cell lymphoma 2; BDNF, brain‐derived neurotrophic factor; BPntase or RnPIP, nucleotide bisphosphate 39‐nucleotidase; CKIa, casein kinase Ia; CREB, cyclic adenosine monophosphate response element‐binding protein; DAG, diacylglycerol; GSK‐3, glycogen synthase kinase 3; HSP, heat‐shock protein; IMPase, inositol monophosphatase; InsP3, inositol triphosphate; LRP6, lipo‐protein related protein 6; MEK, Ras/Raf/MAPK; PKA, protein kinase A; PKC, protein kinase C; RSK, ribosomal S6 kinase; VEGF, vascular endothelial growth factor; Wnt, wingless‐related integration site.

Lithium's therapeutic action is extremely complex, involving multiple effects on gene secretion, neurotransmitter or receptor mediated signaling, signal transduction processes, circadian modulation, as well as ion transport (Dudev et al., 2019; Pisanu et al., 2016). It is worth noting that lithium directly blocked GSK‐3 via the stimulation of serine phosphorylation resting in the activation of Akt or protein kinase A (PKA) and protein kinase C (PKC) (Figure 3) (Ciftci et al., 2020). Also, blockade of GSK‐3 by lithium triggered the expression of numerous neuroprotective as well as neurotrophic proteins, like heat‐shock protein 70 (HSP70), brain‐derived neurotrophic factor (BDNF), as well as Bcl‐2 (Figure 3) (Liang & Chuang, 2007; Ren et al., 2003).

Furthermore, blockade of GSK‐3 by lithium facilitated the stimulation of cell survival transcription factors, such as cyclic adenosine monophosphate response element‐binding protein (CREB), activator protein 1 (AP‐1), β‐catenin, as well as HSP1 and downregulated proapoptotic protein such as p53 and Bcl‐2‐associated X protein (Bax) (Figure 3) (Bijur & Jope, 2000; Chiu & Chuang, 2010). Thus, GSK‐3 is a potent in vivo regulator of cell apoptosis. Additionally, GSK‐3 was capable of inducing neuronal death, which was triggered via prion proteins, p53‐induced apoptosis, as well as amyloid‐beta toxicity (Hu et al., 2009; Watcharasit et al., 2002). Additionally, blockade of GSK‐3 by lithium attenuated TBI‐induced depressive behavior in a mouse model of TBI (Figure 3) (Shapira et al., 2007). Moreover, blockade of GSK‐3 by lithium inhibited the stimulation of p21, a p53 target as well as Bax and Cyto C release as well as caspase stimulation following injury (Figure 3) (Prickaerts et al., 2006).

Lithium expressively augmented hippocampal‐dependent learning as well as memory and decreased hippocampal CA3 neuron loss 5 days post‐injury via GSK‐3 inhibition (Dash et al., 2011). Also, GSK‐3 exists as a fragment of a destruction complex made up of the scaffold protein Axin, the tumor suppressor adenomatous polyposis coli (APC), as well as casein kinase Ia (CKIa) that binds to, and modulates, β‐Catenin‐mediated gene secretion in the absence of wingless‐related integration site (Wnt) (Figure 3) (Grimes & Jope, 2001). Furthermore, binding of Wnt to lipo‐protein related protein 6 (LRP6) receptor complex triggered translocation of GSK‐3 from the cytoplasmic where it binds to and phosphorylates LRP6 into the plasma membrane, resulting in a decreased phosphorylation of β‐catenin as well as reduced proteosomal degradation (Figure 3) (Niehrs & Shen, 2010).

It is worth noting that the translocation of GSK‐3 was associated with a transient but substantial upsurge in Ser^1490^ phosphorylation was observed when the phosphorylation of LRP6 was examined in hippocampal extracts following TBI (Dash et al., 2011). Similarly, the augmented LRP6 phosphorylation triggered a substantial reduction in GSK‐3‐dependent β‐Catenin phosphorylation, which was consistent with augmented Wnt signaling after TBI (Figure 3) (Dash et al., 2011). Thus, Wnt, a fundamental signaling pathway in the modulation of GSK‐3 activity via lithium, is capable of regulating GSK‐3 these activities following TBI (Figure 3) (Jope et al., 2007; Jope & Johnson, 2004)

Notably, Akt is key signaling pathways are capable of modulating GSK‐3 activity via distinct mechanisms (Chiu et al., 2013). However, Akt was unable to prevent TBI‐induced apoptotic cell death, although an increase in Akt activity was detected following TBI (Ciftci et al., 2020). TBI stimulated apoptogenic proteins like caspase proteins as well as stress‐activated kinase c‐Jun N‐terminal kinase (JNK) (Kaidanovich‐Beilin et al., 2004). Also, PI3K as well as Akt modulated the JNK/stress‐activated protein kinase (SAPK) signaling pathway via multiple mechanisms following TBI (Ciftci et al., 2020; Polter et al., 2010). Also, the JNK/SAPK pathway, which is composed of mitogen‐activated protein kinase (MAPK), was associated with stress‐induced cell death (Chiu et al., 2013; Roh et al., 2005).

It is worth noting that TBI triggered the stimulation of members of the MAPK family such as ERK 1/2 and JNK (Ciftci et al., 2020). Furthermore, lithium augmented the phosphorylated Akt and ERK 1/2 levels as well as p38 via GSK‐3α/β following TBI (Ciftci et al., 2020). Specifically, acute lithium treatment augmented Akt, ERK1/2 levels, and GSK‐3 α/β phosphorylations, while prophylactic treatment did not show similar effects on these proteins (Ciftci et al., 2020). Also, lithium effect on GSK‐3β /MEK(Ras/Raf/MAPK)/ERK pathway resulted in a secondary blockade of this kinase via the ribosomal S6 kinase (RSK) (Figure 3) (Son et al., 2003). Moreover, lithium regulation of MEK/ERK/AKT signaling pathway stimulated neuronal function, synaptic plasticity, as well as neuronal survival (Figure 3) (Grewal et al., 1999; A. J. Silva, 2003).

Lithium‐triggered neurite outgrowth in N2A cells was modulated via crosstalk between MEK/ERK as well as PI3K/AKT pathways but most prominently it requires the stimulation of the MEK/ERK signaling pathway (Figure 3) (Wang et al., 2011). Also, inhibition of GSK‐3 by lithium triggered a downregulation of PP1 activity, which resulted changes in kinase/phosphatase balance at Ser 9 position and subsequent hyperphosphorylation at Ser 9 (Figure 3) (Ma & Zhang, 2003; Schepetkin et al., 2022). It is worth noting that lithium indirectly inhibited PKC activity via the blockade of inositol monophosphatase (IMPase) activity, resulting in a decrease in production of diacylglycerol (DAG) as well as inositol triphosphate (InsP_3_) (Figure 3) (Atack et al., 1992). Also, lithium was able to noncompetitively block nucleotide bisphosphate 39‐nucleotidase (BPntase or RnPIP) (Figure 3) (Dollins et al., 2021; Spiegelberg et al., 1999). Furthermore, lithium was capable of inhibiting stress‐induced decrease in vascular endothelial growth factor (VEGF) levels, resulting in the stimulation both neurogenesis as well as angiogenesis (Figure 3) (R. Silva et al., 2007).

Notably, Aβ was elevated in the CSF of patients with TBI, and postmortem human studies also revealed similarly Aβ deposits in TBI patients (Olsson et al., 2004; Uryu et al., 2007). Similarly, GSK‐3 augmented Aβ production via the modulation of APP cleavage following TBI (Phiel et al., 2003). Also, lithium was capable of attenuating Aβ concentrations in vitro and in vivo via the blockade of GSK‐3 following TBI (Figure 3) (Phiel et al., 2003). Furthermore, lithium suppressed TBI‐induced β‐secretase oversecretion and attenuated Aβ levels via blockade of β‐secretase. It is worth noting that BACE1 concentrations were augmented after TBI in human patients and rodent models of TBI (Figure 3) (Loane et al., 2009; Uryu et al., 2007). Moreover, lithium completely inhibited the upsurge BACE1 expression following TBI (F. Yu, Zhang, et al., 2012).

### 2.6 Lithium and neuroprotection

Neuroprotection is the mechanisms as well as strategies used to defend the CNS against injury triggered by both acute as well as chronic neurological disorders (Pisanu et al., 2016; Won & Kim, 2017). Notably, the hippocampal DG has been implicated in neurogenesis as well as behavioral modulation that is associated with anxiety as well as depression in rodents (Cao et al., 2023; Javadapour et al., 2010). Furthermore, injurious conditions often induced alterations in neuroplasticity, resulting in the distraction of synaptic communications in neuronal circuits as observed in the pathophysiology of TBI (Ng & Lee, 2019). Also, the ipsilateral corpus callosum area comprises both myelinated as well as unmyelinated fibers, which normally demonstrate anomalies in TBI patients (Rutgers et al., 2008).

Lithium pretreatment following CCI brain attenuated edema, hippocampal neurodegeneration, as well as loss of hemispheric tissues, and enhanced memory and spatial learning following TBI (Zhu et al., 2010). Additionally, lithium mitigated synaptic dysfunction as well as augmented synaptic proteins associated with dopamine synthesis, secretion, as well as receptor binding in the striatum after TBI (Carlson & Dixon, 2018). Notably, this resulted in a reversal of the neuroplastic impairments related to TBI (Shim et al., 2012). Also, lithium improved spatial learning and memory during Morris's water maze test and well as Y‐maze test. Furthermore, lithium treatment in both acute/prophylactic as well as chronic time resulted in a reduction in the infarct volume in TBI. Specifically, pretreatment with lithium alleviated depressive behavior in cases of mild TBI in mice (Shapira et al., 2007).

Moreover, post‐injury treatment with lithium resulted in a substantial reduction of the TBI induced lesion volume (Strakowski et al., 1999). Additionally, chronic preinjury treatment followed by post‐injury treatment with lithium reduced the lesion volume in a mouse model of TBI (R.‐W. Chen & Chuang, 1999). Specifically, TBI induced a depressive behavior, which was obvious even only after 24 h post‐injury (Kim et al., 2009). Also, lithium was capable of preventing TBI induced depression via the blockade of GSK‐3 (Kim et al., 2009). Notably, lithium treatment in mild TBI triggered protection via the activation of hippocampal AKT phosphorylation as well as blockade of phosphorylation at Ser9 of GSK‐3β and a build up of downstream β‐catenin (Figure 3) (Leeds et al., 2014). Also, 14‐day pretreatment with lithium attenuated IL‐1β secretion in a mouse model using CCI to produce moderate TBI (Figure 1) (Leeds et al., 2014; Zhu et al., 2010).

Furthermore, combination treatment with lithium and etanercept, a TNF‐α inhibitor, attenuated TNF‐α as well as GFAP concentrations and alleviated neuronal degeneration, edema, as well as axonal swelling in rats with diffuse severe TBI model (Ekici et al., 2014; Leeds et al., 2014). Additionally, massive hippocampal neuronal death with associated cognitive impairment was observed in transgenic TBI mice overexpressing mutant APP with elevated Aβ level (Figure 3) (Smith et al., 1998). Thus, lithium improved spatial learning as well as memory via the attenuation of Aβ level (F. Yu, Zhang, et al., 2012). Also, lithium induced protection against neurodegeneration as well as cognitive deficits via Tau phosphorylation in TBI (Figure 1) (F. Yu, Zhang, et al., 2012). Moreover, lithium enhanced motor performance in following TBI, but the degree of recovery was injury severity dependent (F. Yu, Zhang, et al., 2012).

## 3 CONCLUSION

TBI triggers multiple short‐ as well as long‐term modifications in neuronal circuits that eventually lead to imbalance of cortical excitation as well as inhibition and lithium is able to normalize the imbalance. Lithium concentrations are more pronounced in the hippocampus, thalamus, neo‐cortex, olfactory bulb, amygdala, and the gray matter of the cerebellum following treatment. Also, neuroinflammation contributes to neurological deterioration following TBI, and lithium is capable of attenuating neuroinflammation as well as neuronal toxicity leading enhance neuronal survival. Moreover, lithium protected the brain from edema, hippocampal neurodegeneration, as well as loss of hemispheric tissues, and enhanced memory and spatial learning after TBI.

## AUTHOR CONTRIBUTIONS

**Seidu A. Richard**: Conceptualization; methodology; writing—original draft; writing—review and editing; software.

## CONFLICT OF INTEREST STATEMENT

The author declares no conflicts of interest.

### PEER REVIEW

The peer review history for this article is available at https://publons.com/publon/10.1002/brb3.3595.

## Data Availability

Data sharing is not applicable to this article as no new data were created or analyzed in this study.
